# Predictors of Infections following Cranioplasty: A Retrospective Review of a Large Single Center Study

**DOI:** 10.1155/2014/356042

**Published:** 2014-10-22

**Authors:** Mario Zanaty, Nohra Chalouhi, Robert M. Starke, Rohan Chitale, Shannon Hann, Cory D. Bovenzi, Mark P. Saigh, Eric W. Schwartz, Emily S. I. Kunkel, Alexandra S. Efthimiadis-Budike, Pascal Jabbour, Richard Dalyai, Robert H. Rosenwasser, Stavropoula I. Tjoumakaris

**Affiliations:** ^1^Division of Neurovascular Surgery and Endovascular Neurosurgery, Department of Neurological Surgery, Thomas Jefferson University Hospital, Thomas Jefferson University, 901 Walnut Street, 3rd Floor, Philadelphia, PA 19107, USA; ^2^Department of Neurological Surgery, University of Virginia, Charlottesville, VA 22908, USA

## Abstract

*Introduction.* The variables that predispose to postcranioplasty infections are poorly described in the literature. We formulated a multivariate model that predicts the risk of infection in patients undergoing cranioplasty. *Method.* Retrospective review of all patients who underwent cranioplasty following craniectomy from January, 2000, to December, 2011. Tested predictors were age, sex, diabetic status, hypertensive status, reason for craniectomy, urgency status of craniectomy, location of cranioplasty, reoperation for hematoma, hydrocephalus postcranioplasty, and material type. A multivariate logistic regression analysis was performed. *Results.* Three hundred forty-eight patients met the study criteria. Infection rate was 26.43% (92/348). Of these cases with infection, 56.52% (52/92) were superficial (supragaleal), 43.48% (40/92) were deep (subgaleal), and 31.52% (29/92) were present in both the supragaleal and subgaleal spaces. The predominant pathogen was coagulase-negative staphylococcus (30.43%) followed by methicillin-resistant *Staphylococcus aureus* (22.83%) and methicillin-sensitive *Staphylococcus aureus* (15.22%). Approximately 15.22% of all cultures were polymicrobial. Multivariate analysis revealed convex craniectomy, hemorrhagic stroke, and hydrocephalus to be associated with an increased risk of infection (OR = 14.41; *P* < 0.05, OR = 4.33; *P* < 0.05, OR = 1.90; *P* = 0.054, resp.). *Conclusion.* Many of the risk factors for infection after cranioplasty are modifiable. Recognition and prevention of the risk factors would help decrease the infection's rate.

## 1. Introduction

Cranioplasty is performed for a blend of medical and aesthetical reasons [1]. While cranioplasty is known to improve neurological outcomes in patients with craniectomy, cranioplasty infection can lead to reoperation, long-term antibiotic use, and significant morbidity [2–8], which eventually may outweigh its benefit. Many reports in the literature aimed to evaluate the risk factors of cranioplasty infection. However, some of their results were contradictory, and the full model remains little elucidated. We aimed to formulate a multivariate model that predicts the risk of graft infection in patients undergoing cranioplasty.

## 2. Method

### 2.1. Design

After receiving the University Institutional Review Board approval, we conducted a retrospective review of all patients who underwent cranioplasty following craniectomy for stroke, subarachnoid hemorrhage, and trauma at our institution in the period from January 2000 to December 2013.

### 2.2. Variables

We tested the following predictors: age, sex, diabetic status, hypertensive status, tobacco use, reason for craniectomy, urgency status of craniectomy (urgent versus elective), location of cranioplasty (convexity, bilateral convexity, bifrontal, and suboccipital), reoperation for hematoma evacuation, hydrocephalus postcranioplasty (documented by a CT scan), cranioplasty material type (autologous versus synthetic), and seizures development after the craniectomy. Patients with CSF leak and those who underwent cranioplasty for infectious etiology were excluded from the study. A multivariate logistic regression analysis was performed.

In addition, we reviewed the results of culture from the purulent material and necrotic debris that were sent for testing. We defined a cranioplasty infection in any case that needed cranioplasty graft removal or in any case in which infection was suspected and antibiotic therapy was administrated for more than 2 weeks (regardless of culture results). Postcranioplasty infection was divided into superficial and deep with respect to galea invasion. Patients who had craniotomy for infectious disease were not included in the study.

### 2.3. Data Analysis

Data are presented as mean and range for continuous variables and as frequency for categorical variables. Analysis was carried out using unpaired *t*-test, chi-square, and Fisher's exact tests as appropriate. Univariate analysis was used to test covariates predictive of cranioplasty site infection. Interaction and confounding were assessed through stratification and relevant expansion covariates. Factors predictive in univariate analysis (*P* < 0.15) [9] were entered into a multivariate logistic regression analysis. *P* values of ≤0.05 were considered statistically significant. Statistical analysis was carried out with Stata 10.0 (College Station, TX).

## 3. Results

### 3.1. Demographic Variables

Three hundred sixty patients met the study criteria. Data analysis revealed a mean age of 49.80 +/− 15.50 years. Males accounted for 51.11% percent of the sample while females accounted for 48.89%. Fifteen-percent of our patients were diabetic, 56.94% were hypertensive, and 46.94% were smokers. The majority of the patients received autologous bone graft (67.22%). The locations of cranioplasty were classified as convexity (91.11%), bifrontal (8.92%), and suboccipital (0.57%).

The proportion of patients who underwent a second operation for hematoma evacuation after cranioplasty was 6.89%. Other postcranioplasty complications were seizures (14.44%) and hydrocephalus (13.61%).

### 3.2. Predictors of Infection

The infection rate was 25.55% (92/360). Of these infected cases, 56.52% (52/92) were superficial (supragaleal) infection and constituted 56.52% (52/92), while deep infection constituted 43.48% (40/92) of the cases. As much as 31.52% (29/92) of the cases had both a supragaleal and a subgaleal space infection. The predominant pathogen was coagulase-negative* Staphylococcus* (30.43%) followed by methicillin-resistant* Staphylococcus aureus* (22.83%), methicillin-sensitive* Staphylococcus aureus* (15.22%),* Propionibacterium acnes* (18.48%), and* Enterobacterium cloacae* (7.61%). Polymicrobial culture made about 15.22% of all cultures (Table 1).

Univariate analysis (Table 2) revealed that increasing age, bilateral convexity cranioplasty (versus suboccipital, bifrontal, and unilateral convexity cranioplasty), diabetes mellitus, hemorrhagic stroke, and postcranioplasty hydrocephalus were predictive of infection. Gender and race did not increase the risk of infection. In addition, hypertension and smoking were not significantly associated with a higher risk of graft infection. Urgent craniectomies did not affect the risk of infection when compared to elective ones. Finally graft material, reoperation for hematoma evacuation, and the development of seizures were not predictors in univariate analysis. In multivariate analysis (Table 3), bilateral convexity cranioplasty, postcranioplasty hydrocephalus, older age (>65), and hemorrhagic stroke remained associated with a higher risk of infection (OR = 15.66; *P* < 0.001; OR = 2.30; *P* = 0.049; OR = 1.26; *P* = 0.050; OR = 8.63; *P* < 0.001, resp.).

## 4. Discussion

Many potential variables were studied in the literature yielding controversial results. Hence, we attempted to test important potential risk factors. The study infection rate is slightly higher than that reported in the literatures, which ranged from 0–2% to 21.4% [10, 11]. We believe that the reason is the definition of infection in our study that was not limited to reoperation. We found that skin organisms, mainly* Staphylococcus* and* Propionibacterium* species, were the dominant pathogens, which is consistent with the findings of J. N. Bruce and S. S. Bruce [12]. The effect timing has on cranioplasty infection is debatable. While some studies reported no difference in the rate of infection between cranioplasty performed within 3 months (early) and more than 3 months (late) after craniectomy, a systematic review [10] that examined 5 studies found that only one of them reported a significantly lower rate of infection in early cranioplasty (<3 months) [11]. The other four studies [2, 13–15] reported a higher rate of infection when cranioplasty was performed early, but statistical significance was not achieved in any of them [10]. The systematic review also did a meta-analysis of the pooled data from the five studies and found no significant difference in infection rate between early and late cranioplasty. Recently, Walcott et al. reviewed 239 cranioplasty cases and found no association between cranioplasty timing and infection development [16]. For all the reasons above and given that cranioplasty seems to aid in healing and fasten the rehabilitation process, the large majority of our patients had received early cranioplasty (<3 months). Cranioplasty was delayed for more than 3 months only if we feel that the patient has not yet fully recovered or if the patient has significant morbidities that can be controlled before intervening again.

### 4.1. Graft Material

Park et al. [17] and Mollman and Haines [18] argued that placement of foreign bodies may increase the risk of postoperative infection. Matsuno et al. found that polymethyl-methacrylate (PMMA) has a higher rate of infection when compared to autologous bone graft [8]. Other studies yielded different results. Titanium mesh was found to have a lower risk of infection than autologous graft [8]. Our series reproduce the finding of many others, suggesting no difference in the infection rate between synthetic and autologous grafts [2, 16].

### 4.2. Demographics

Similar to recent studies, we found no significant association of age, gender, diabetes, and therapeutic indication (SAH, trauma, and ischemic stroke) with postcranioplasty infection [19, 20]. We found that reoperation for hematoma showed a trend of higher infection risk but was not statistically significant in multivariate analysis. While multiple procedures have been found to increase the risk of infection in the literature [8, 16, 19], Cheng et al. [2] did not find any significant association. Walcott et al. [16] analysis identified therapeutic indication for stroke as significantly associated with the development of cranioplasty infection. We found hemorrhagic stroke to be predictive of infection in multivariate analysis; a possible explanation would be the shared risk factor between stroke and infection, such as diabetes and smoking [21, 22]. Both diabetes [23] and smoking [24] are well known to increase the risk of surgical site infection, but these findings are not always consistent, as our study showed that diabetes and smoking are not reliable predictors of graft infection. One reason would be the heterogeneity of diabetic patients in terms of blood sugar control and the lack of categorizing smokers between current and former. Such variations might change the risk of developing postsurgery infection [25, 26]. Walcott and colleagues also reported that patient age, location of cranioplasty, presence of an intracranial device, bone flap preservation method, cranioplasty material, and booking method were not predictive of the development of cranioplasty infection [16]. Other parameters examined in the literature were subgaleal fluid, on which the results were divisive [19, 20], and the presence of neurological deficits before cranioplasty, which was found to increase the infection rate [11]. Poor nutritional status has been shown to increase the surgical infection risk [27–29] but was not studied as a predictor of cranioplasty infection.

### 4.3. Controlling the Risk

Some factors (diabetes, hypertension) that predict infection should be aggressively managed. We advise delaying the intervention until improvement in the patient's comorbidity and blood sugar level and blood pressure are adequately controlled. Unfortunately, many significant predictors, notably bilateral convexity cranioplasty and older age, remain uncontrollable. Upcoming studies should consider correlating the amount of bone removed with the infection rate; perhaps a slighter amount of bones could provide adequate decompression while allowing a lower risk of graft infection. We found it interesting that a significant portion of infection was attributed to* S. epidermidis, *which could reflect potential contamination. Perhaps paying careful attention and developing better sterile technique and preservation methods may substantially decrease the rate of graft infection. Furthermore,* Staphylococcus aureus *infection contributed to another 30% of the infection rate. We suggest that future studies investigate whether postoperative antibiotics for a prolonged period would decrease the rate of infection, particularly in MRSA and MSSA carriers. Working on developing a checklist may be worthwhile to perhaps help decrease infections in the future. Another potential method of controlling the infection would be to prescribe a course of antibiotics for P acnes carrier before the surgery. This would be an interesting matter for future studies to interrogate, given the paucity of literature on antimicrobial management for patients undergoing cranioplasty.

## 5. Limitations

The main limitation of the study is the retrospective design. In addition, one of the limitations was that the stratification did not account for former and current smokers, as well as controlled and uncontrolled diabetes. We considered that such extensive stratification would render the samples size too small for robust statistical analysis.

## 6. Conclusion

Much of the literature focused on patient specific factors as a major predisposition to infection, the majority of which are observational and lack high-quality evidence [10]. Our final model showed that the most significant predictors of postcranioplasty infection are hydrocephalus, bilateral convexity location, older age, and hemorrhagic stroke. Therefore, controlling stroke risk factor and preventing the development of one complication might decrease the risk of cranioplasty infection. Our results may help the neurosurgeon identify high-risk patients in the future.

## Figures and Tables

**Table 1:** Culture results.

Pathogen	Proportion
Staphylococcus aeurus	
Methicillin-Resistant	22.83%
Methicillin-Sensitivity	15.22%
Coagulase Negative Staphylococcus	30.43%
Pseudomonas	
Enterobacterium	
*Cloacae *	7.61%
*Aerogenes *	1.84%
Acinetobacter species	1.84%
Propionibacterium *acnea *	18.48%
Klebsiella *pneumonia *	1.84%
Streptococcus *agalactia *	1.84%
Corynebacterium species	1.08%
Polymicrobial culture	15.22%

**Table 2:** Univariate analysis for predictors of infection.

Predictors	Odds Ratio (OR)	*P* value
Diabetes mellitus	OR = 2.63	*P* < 0.01
Bilateral Convexity cranioplasty	OR = 10.04	*P* < 0.01
Hemorrhagic stroke	OR = 7.26	*P* < 0.01
Reoperation for hematoma	OR = 2.56	*P* = 0.051
Post-cranioplasty hydrocephalus	OR = 3.11	*P* < 0.01
Gender	OR = 0.94	*P* = 0.81
Age	OR = 2.77	*P* = 0.049

**Table 3:** Multivariate analysis for predictors of infection.

Predictors	Odds Ratio (OR)	*P* value
Bilateral Convexity cranioplasty	OR = 15.66	*P* < 0.01
Hemorrhagic stroke	OR = 8.63	*P* < 0.01
Hydrocephalus	OR = 2.30	*P* = 0.05
Older Age (>65 years)	OR = 1.26	*P* = 0.050
